# Kawasaki disease before and during the COVID-19 pandemic: a single-center comparative study in Switzerland

**DOI:** 10.1186/s12887-024-05115-0

**Published:** 2024-10-08

**Authors:** Justine Epitaux, Nicole Sekarski, Sabrina Bressieux-Degueldre

**Affiliations:** 1 Faculty of Biology and Medicine, University of Lausanne, Rue du Bugnon 21, Lausanne, 1011 Switzerland; 2 Department of woman-mother-child, Unit of Pediatric Cardiology, Lausanne University Hospital and University of Lausanne, Rue du Bugnon 21, Lausanne, 1011 Switzerland; 3 Department of woman-mother-child, Unit of Pediatric Cardiology, Lausanne University Hospital, Rue du Bugnon 21, Lausanne, 1011 Switzerland

**Keywords:** Kawasaki disease, COVID-19 pandemic, Incidence, Coronary artery outcome.

## Abstract

**Background:**

Kawasaki disease is a rare systemic inflammatory syndrome that mainly affects children under five years of age and is the first cause of pediatric acquired cardiovascular disease. The pathogenesis is complex and a viral trigger is suspected, as well as genetic susceptibility. Multiple studies around the world have shown a decrease in the incidence of Kawasaki disease and have hypothesized that the different sanitary measures enforced in each country during the pandemic period could be responsible to a certain extent. The aim of this study is to evaluate the effects of the COVID-19 pandemic on the disease’s incidence, defining characteristics, coronary artery outcomes and management in a tertiary center in Switzerland.

**Methods:**

This study is a retrospective analysis of children who have been diagnosed with Kawasaki disease that compares clinical, laboratory, SARS-CoV-2 exposure, and echocardiographic data as well as treatments before (January 1st 2017 to February 24th 2020) and during (February 25th 2020 to December 31st 2022) the COVID-19 pandemic in Switzerland. Statistical significance of differences in the compared parameters was assessed.

**Results:**

Of the 90 patients included, 31 belonged to the first group and 59 belonged to the second group. There was a statistically significant (*p* < 0.05) increase in incidence during the pandemic period (5.91/100,000 children) of 88% compared to the pre-pandemic period (3.14/100,000 children). A lesser seasonal variation was observed during the pandemic. 30% of the patients in the pandemic group had an exposure to SARS-CoV-2. There was no other notable difference in demographic factors, clinical presentation, coronary outcome or administered treatment.

**Conclusions:**

To the best of our knowledge, this is the first prolonged European study comparing Kawasaki disease before and during the COVID-19 pandemic. There was a significant increase in incidence in Kawasaki disease during the COVID-19 pandemic. In contrast, studies done in Japan, South Korea and the USA have shown a decrease in incidence. Differences in methodologies, genetics, ethnicities, environments, microbiome-altering behaviors, sanitary measures and SARS-CoV-2 spread are factors that should be considered. Further studies analyzing the differences between countries with increased incidence of Kawasaki disease could help better understand the relevance of such factors and provide more insight into the etiologies of this particular disease.

## Background

Kawasaki disease (KD) is a rare systemic inflammatory syndrome that mainly affects children under five years of age and is the first cause of pediatric acquired cardiovascular disease [1]. The standard treatment for KD consists of high doses of intravenous immunoglobulins (IVIG) and acetylsalicylic acid (ASA) within ten days from disease’s onset, which has been shown to diminish the cardiac sequelae [2].

As the pathogenesis of KD is complex, its origin remains unknown, although an infectious viral trigger is suspected [3]. This is particularly the case because of the epidemic-like pattern of the disease’s presentation as well as its seasonality that has a peak during January [4, 5]. Additionally, genetic susceptibility is suspected due to the far greater prevalence of the disease in children of Asian descent, particularly Japanese descent, compared to the rest of the world, in addition to higher incidences of KD if a sibling has already been affected [5]. It is believed that both aspects contribute to the development of an inappropriate immunological response and therefore the systemic inflammation.

A previous prospective national study between 2013 and 2017 defined the incidence of KD in Switzerland as 3.1/100,000 children [6].

With the arrival of the COVID-19 pandemic, a new post-infectious inflammatory syndrome appeared in children. It was initially described as a Kawasaki-like disease and is now named Multisystem Inflammatory Syndrome in Children (MIS-C), or Paediatric Inflammatory Multisystem Syndrome temporally associated with SARS-CoV-2 (PIMS-TS). This syndrome and KD share similarities in some aspects, such as the affected population as well as their clinical presentation [7, 8]. Because of this resemblance, overlaps may have occurred in the diagnoses and management of KD and MIS-C, especially in the early pandemic era.

The aim of this study is to evaluate the effects of the COVID-19 pandemic on the disease’s incidence, defining characteristics, coronary artery outcomes and management in a tertiary center in Switzerland.

## Methods

This study is a retrospective analysis of children who were diagnosed with KD that compares epidemiological, demographic, clinical, laboratory, including SARS-CoV-2 exposure, and echocardiographic data for coronary artery outcomes, as well as treatments before and during the COVID-19 pandemic in Switzerland.

All patient records with a diagnosis of KD referred to the Pediatric Cardiology Unit of the Lausanne University Hospital between January 1st 2017 and December 31st 2022 were reviewed. KD diagnosis was based on the American Heart Association (AHA) guidelines [1]. All patients were underneath the age of 18 years at the time of diagnosis and were excluded if fever was explained by a diagnosis other than KD, or if their medical file contained insufficient data.

The patients were categorized into two groups depending on the date of their KD diagnosis. The first group included patients from the pre-pandemic period from January 1st 2017 to February 24th 2020. The second group included patients having been diagnosed during the pandemic, which began when the first COVID-19 infection was confirmed in Switzerland [9], from February 25th 2020 to December 31st 2022. The COVID-19 infection kept its pandemic status until 2023, although sanitary measures varied throughout that period [10].

The following data was collected: demographic factors such as age at the time of diagnosis, sex and ethnicity, clinical presentation (typical versus atypical Kawasaki disease in accordance with the AHA’s guidelines) and treatment administered. For each patient, the most abnormal laboratory value within their acute KD period was selected.

SARS-CoV-2 virus exposure was considered positive if the patient had positive SARS-CoV-2 serology for the virus, a positive nasopharyngeal polymerase chain reaction (PCR) for SARS-CoV-2, or a positive history of SARS-CoV-2 exposure within 4 weeks prior to the onset of symptoms.

Echocardiography reports were reviewed for coronary artery involvement (CAI) (number and size of coronary artery aneurysms) and, in cases of involvement, z-scores for coronary artery diameters were calculated [11]. In accordance with the AHA guidelines, a dilatation was defined as a z-score ≥ 2 and < 2.5, and an aneurysm was defined as a z-score ≥ 2.5 (small ≥ 2.5 and < 5, medium ≥ 5 and < 10 or absolute diameter < 8 mm, giant ≥ 10 or absolute diameter ≥ 8 mm). If a patient presented with multiple locations of CAI, only the largest one was taken into account and listed.

KD incidence was calculated using cantonal registries of the pediatric population numbers per year [12].

Statistical significance of differences in parameters between the two groups were assessed by using Student’s T-test (two-tailed) for continuous variables and Pearson’s χ2 test for categorical variables. Statistical significance was set at a P-value of < 0.05.

## Results

Between January 1st 2017 and December 31st 2022, 99 patients were referred to our center with a diagnosis of KD. 9 patients were excluded because they either had their KD diagnosis rejected by another diagnosis or because their medical files contained insufficient data. Of the 90 patients included, 31 belonged to the first group (pre-pandemic) and 59 belonged to the second group (during the pandemic).

The epidemiological, demographic, clinical and laboratory findings are summarized in Table 1.

**Table 1:** Clinical parameters: This table shows the epidemiological, demographic, clinical and laboratory findings of the patients, comparing before and during the COVID-19 pandemic. Variables are expressed as number_/total_ (%), mean (± standard deviation), or median (interquartile range). P-values written in bold indicate statistical significance

	Pre-COVID	During COVID	*P*-value
Number of patients	31	59	**0.004**
**Epidemiological and demographic findings**			
Age at diagnosis (years)	3.00 (1.25–4.00)	2.50 (1.00–4.00)	0.975
Children under 1 year old	8_/31_ (25.8%)	16_/59_ (27.1%)	0.909
Males	18_/31_ (58.1%)	31_/59_ (52.5%)	0.712
**Clinical findings**			
Days from first day of fever to diagnosis	7.00 (6–10)	7.00 (5–9)	0.089
Duration of fever (days)	7.00 (6–10)	8.00 (6–10)	0.603
Complete presentation	21_/31_ (67.7%)	31_/59_ (52.5%)	0.367
**Main symptoms**			
Bilateral conjunctival injection	26_/30_ (86.7%)	43_/59_ (72.9%)	0.572
Changes in lips and the oral cavity	28_/30_ (93.3%)	52_/59_ (88.1%)	0.917
Cervical lymphadenopathy	17_/30_ (56.7%)	27_/59_ (45.8%)	0.558
Extremity changes	17_/30_ (56.7%)	28_/58_ (48.3%)	0.638
Polymorphous rash	27_/30_ (90.0%)	44_/58_ (75.9%)	0.525
**Other symptoms**			
Gastrointestinal symptoms	10_/31_ (32.3%)	20_/59_ (33.9%)	-
Neurological symptoms	3_/31_ (9.7%)	4_/59_ (6.8%)	-
Articular symptoms	1_/31_ (3.2%)	2_/59_ (3.4%)	-
**Laboratory findings**			
Sedimentation rate (mm/h)	80.89 (± 28.23)	70.89 (± 32.08)	0.240
Thrombocytes (G/l)	537.31 (± 334.58)	451.92 (± 264.92)	0.210
Leucocytes (G/l)	14.97 (± 7.03)	14.71 (± 6.75)	0.871
ALAT (U/I)	74.61 (± 168.64)	54.89 (± 100.32)	0.547
CRP (mg/l)	118.89 (± 108.84)	124.32 (± 87.75)	0.807
Hemoglobin (g/l)	104.04 (± 10.23)	99.80 (± 11.77)	0.115
Albumin (g/l)	33.80 (± 6.23)	31.52 (± 6.71)	0.208

**Fig. 1:**
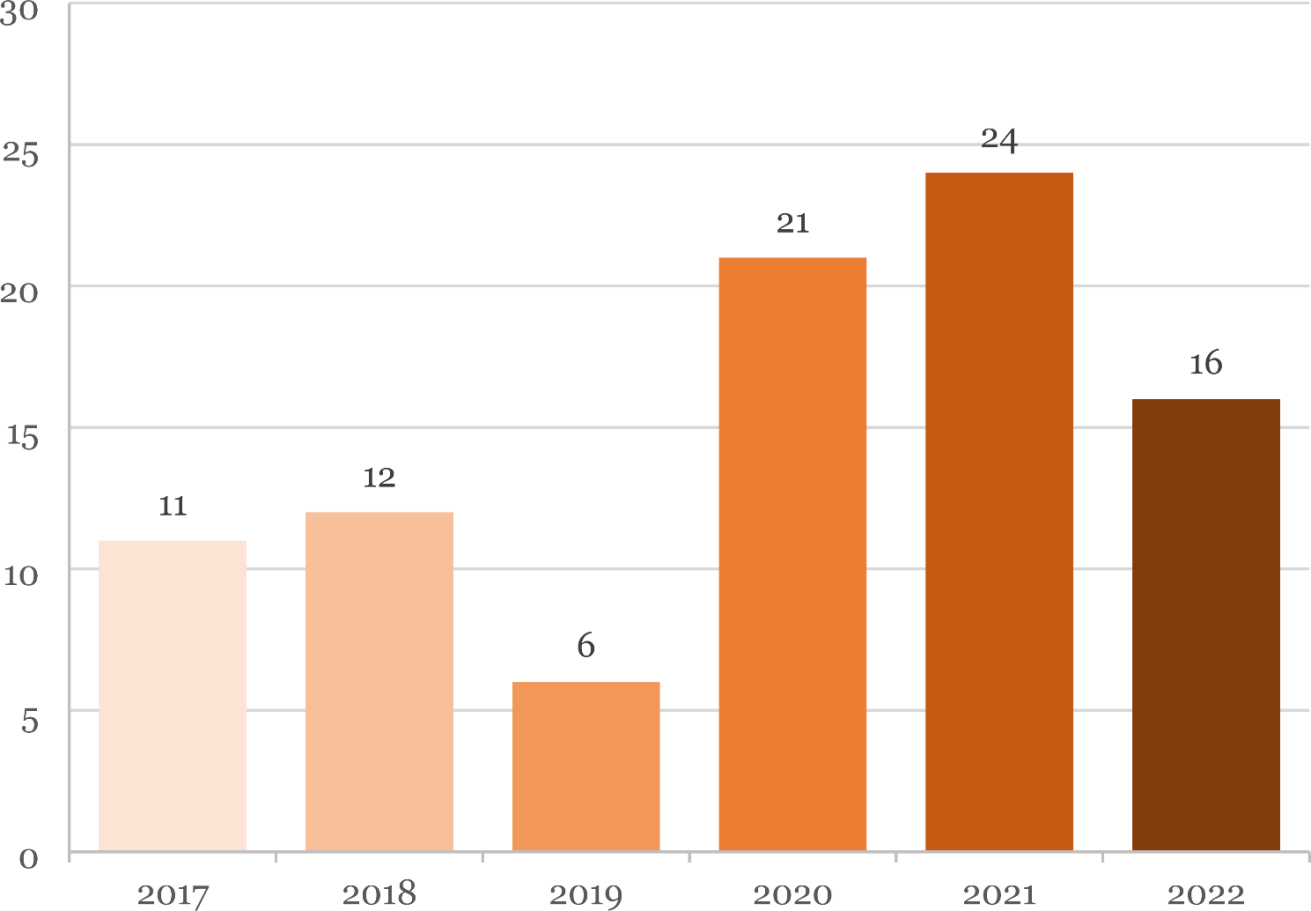
KD cases per year: This figure shows the number of KD cases diagnosed per year. It is to be noted that 2 of the cases counted in the year 2020 belong to the pre-pandemic group

There is a statistically significant increase in KD incidence during the pandemic period of 88% (5.91/100,000 children) compared to the pre-pandemic period (3.14/100,000 children) (Table 1; Fig. 1).

**Fig. 2:**
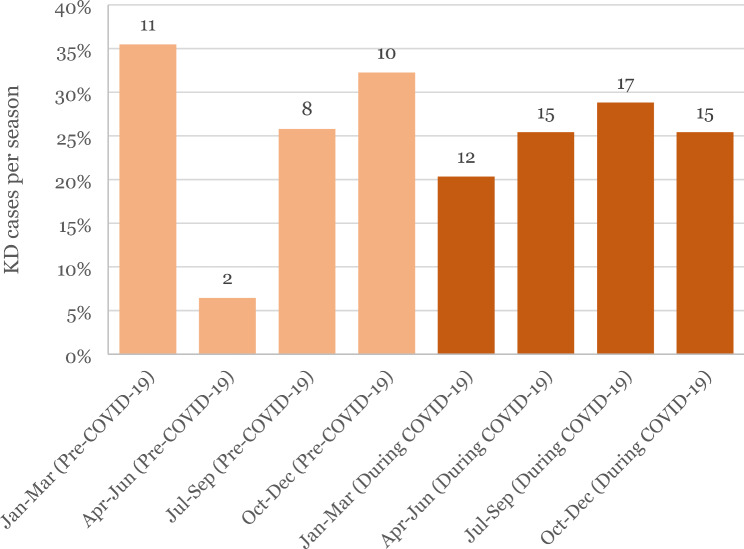
Seasonal distribution of KD cases: This figure shows the number and relative percentage of KD cases per year quarter

Figure 2 illustrates the seasonality of KD incidence. It can be noted that in the pre-pandemic group, there is a marked seasonal variation between the quarters, whereas in the pandemic group, seasonality is less prominent.

Age and sex have not varied, and a greater proportion of male patients remains (Table 1). Ethnicity was not included in the table due to a lack of information.

The two most prevalent symptoms remained changes in the lips and oral cavity as well as the polymorphous rash. Overall, there was no significant difference in clinical presentation. The complete or classical presentation of KD remained the most prevalent form in the two groups. Importantly, time from first day of fever to diagnosis remained unchanged with a median of 7 days in both groups.

There was no notable variation in the laboratory values between the two groups (Table 1).

Of the 59 patients in the pandemic group, 52 had available data concerning SARS-CoV-2 virus exposure and 16 (30.8%) were positive. Of those, 1 patient (6%) had a positive PCR, 4 (25%) had a positive history of SARS-CoV-2 exposure within the last 4 weeks (3 of which had a positive serology), and 11 (69%) only had a positive SARS-CoV-2 serology.

**Fig. 3:**
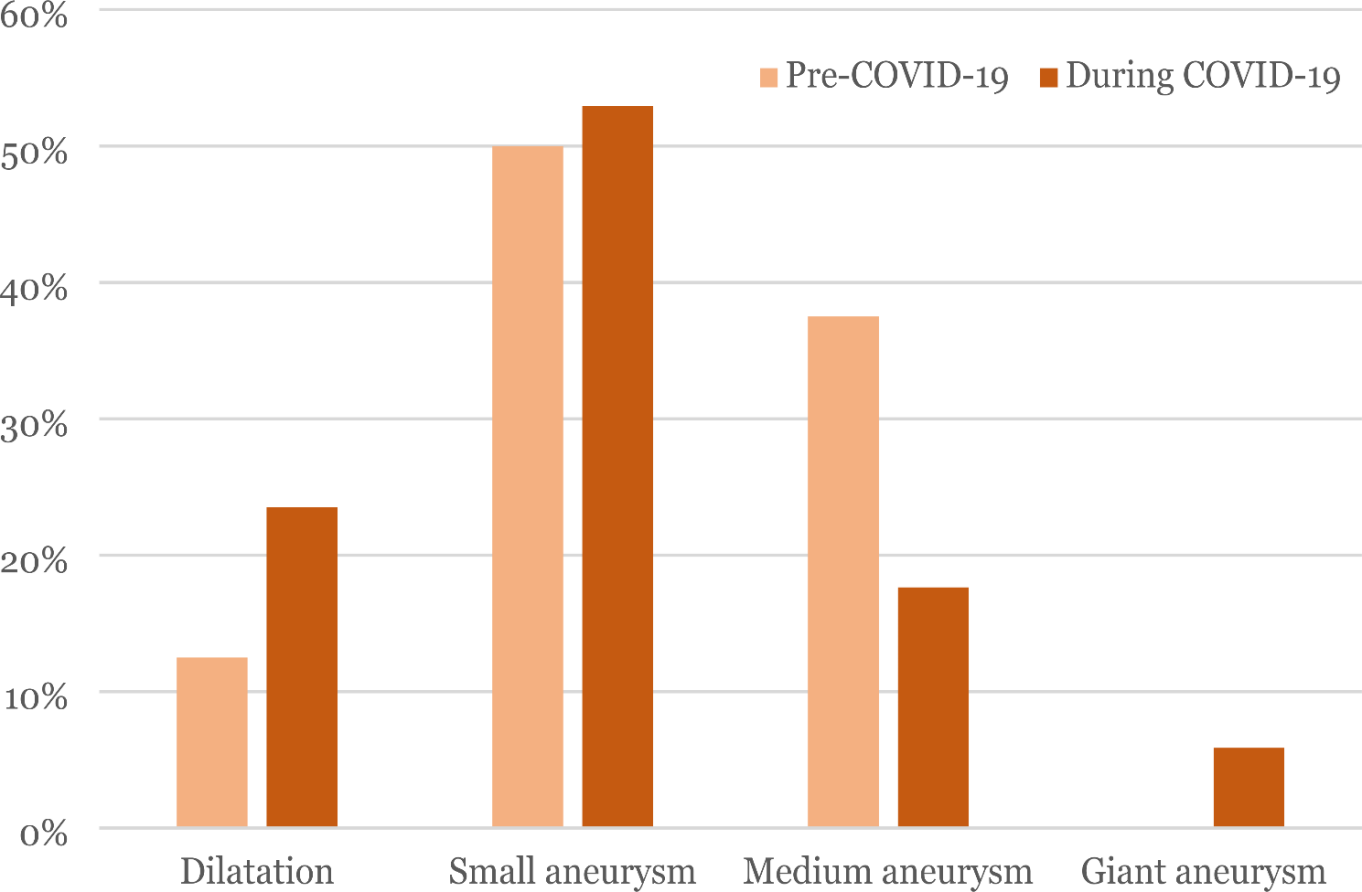
Coronary artery lesion size: This figure shows the percentage of coronary artery lesion according to size, comparing before and during the COVID-19 pandemic

The coronary outcomes remained unchanged before and during the COVID-19 pandemic, with no obvious changes in the proportion of patients having a coronary artery lesion, nor a difference in its severity, with small aneurysms being the most prevalent lesion (Table 2; Fig. 3). Only one patient diagnosed during the pandemic presented with a giant aneurysm. The incidence of aneurysms remained stable.

**Table 2:** Coronary artery outcomes: This table shows the coronary artery outcomes of the patients, comparing before and during the COVID-19 pandemic. Number of patients with at least one coronary artery involvement. Variables are expressed as number (%) or mean (± standard deviation)

Coronary artery outcomes	Pre-COVID	During COVID	*P*-value
Number of patients	31	59	
Coronary artery involvement	8 (25.8%)	17 (28.8%)	0.797
Coronary artery dilatations	1 (3.2%)	4 (6.8%)	-
Coronary artery aneurysms (CAA)	7 (22.6%)	13 (22.0%)	-
CAA Z-score	5.03 (± 2.51)	4.47 (± 3.72)	0.703

**Fig. 4:**
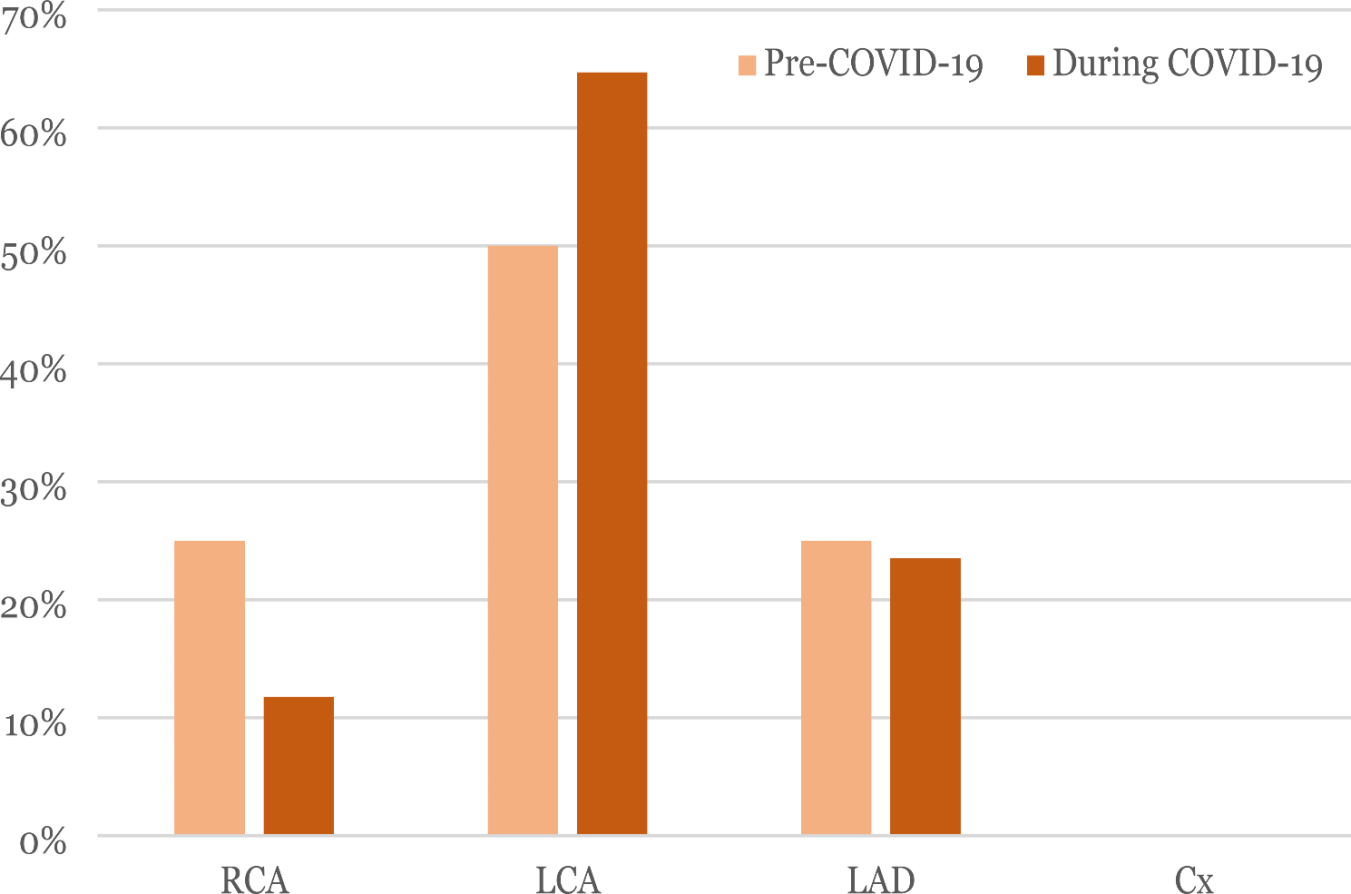
Coronary artery lesion location: This figure shows the percentage of coronary artery lesion according to its location, comparing before and during the COVID-19 pandemic. RCA: right coronary artery. LMCA: left main coronary artery. LAD: left anterior descending artery. Cx: Left circumflex artery

Figure 4 illustrates the location of the most severe coronary artery lesion. Both before and during the pandemic, the most prevalent location was the left main coronary artery (LMCA).

The treatments patients received are summarized in Table 3. The majority of patients were given the standard treatment recommended by the AHA’s international guidelines, which is IVIG administered within the first 10 days of the illness and ASA in doses between 30 and 100 mg/kg/day.

**Table 3:** Treatments administered: This table shows the treatments that were administered to the patients, comparing before and during the COVID-19 pandemic. Variables are expressed as number (%). ASA: acetylsalicylic acid

Treatment	Pre-COVID	During COVID	*P*-value
Number of patients	31	59	
IVIG	28 (90.3%)	59 (100%)	0.657
High dose ASA	27 (87.1%)	56 (94.9%)	0.771
Corticosteroids	0 (0.0%)	4 (6.8%)	-

The 3 patients who did not receive IVIG as part of their treatment were diagnosed retrospectively after the acute phase of the disease had been resolved. Of the 7 patients who did not receive ASA at high doses, 5 were diagnosed after the 10th day of illness and were therefore only given ASA at antiaggregant doses. Of the 2 remaining patients, 1 was treated with antiaggregant doses because of the initial differential diagnosis with MIS-C. Finally, of the 4 patients in the pandemic group who were also given corticosteroids (CS), 3 received CS instead of a second dose of IVIG for IVIG-resistant KD, and 1 received CS because of the presence of a giant aneurysm.

## Discussion

Our study, which was based in a tertiary care center in Switzerland, has found an almost 90% increase in the incidence of Kawasaki disease after the beginning of the COVID-19 pandemic (3.14/100,000 before, 5.91/100,00 during). The pre-pandemic incidence is consistent with an epidemiological Swiss study conducted from 2013 to 2017 [6].

In contrast, studies in the United States, Korea, Japan and Iran revealed a 28–60% decrease in KD incidence after the beginning of the pandemic [13–18]. All have cited the sanitary efforts mitigating the propagation of the SARS-CoV-2 virus and, by extension, other airborne viruses, or the decrease in air pollution as potential explanations for such findings.

The incidence decreases cited above [13, 15–18] were investigations that ended in 2020, which means that their pandemic incidence was not collected over the span of one complete year and therefore could not fully take into account the seasonal variability of KD, as discussed below. Conversely, our study spanned over three pandemic years and could therefore better appreciate this variability.

For the Asian studies, the inclusion criteria were not similar to ours as they defined KD according to the Japanese guidelines, in which fever is not an obligatory symptom for complete KD and rash must include redness at the site of BCG (Bacille Calmette-Guèrin) inoculation [19], or according to the International Classification of Diseases, in which there are no specific diagnostic criteria. Additionally, it is well accepted that genetic susceptibility to KD is different in Asian populations than in Western ones and, despite their proportional decrease in incidence, theirs remain above ours (18.8/100,000 compared to our 5.9/100,000) [15].

However, differences in methodologies do not provide a satisfactory explanation of the difference in incidence our study has found compared to the other countries. The sanitary measures and decrease in air pollution caused by the pandemic should also be true for the Swiss population, yet KD incidence increased in Switzerland, which leads us to deduce that alone, those circumstances do not suffice to explain such differences in incidence variations. Other well-accepted KD triggers, such as genetics, race and environment, could influence the impact that the aforementioned conditions had on KD onset. Furthermore, studies focusing on the immunological aspects of KD pathogenesis have introduced concepts such as an association with gut dysbiosis [20–22] and comparisons with other infection-related immune-mediated diseases [22]. It has been shown that the use of microbiome-altering antibiotics typically worsens KD manifestations [21] and that poorer gut health is associated with inflammation in KD [23]. Therefore, when comparing KD before and during the COVID-19 pandemic, it is important to also take into account changes in diet, antibiotic use and behaviors leading to the diversification of children’s microbiomes [24, 25] that may have varied differently between countries.

Three other European studies described an increase in incidence during the early COVID-19 pandemic period in Italy, France and Spain [26–28]. All three studies gathered the pandemic KD cases over the span of only a couple of months (between 2 and 3 months) and had reported a 2- to 30-fold increase in KD incidence. In the French and Italian studies, although KD cases were also defined by the American Heart Association’s criteria, it must be noted that the majority of the patients (80% and 90%) had a positive exposure to the SARS-CoV-2 virus (serology, nasopharyngeal swab PCR or close contact with someone infected). Later, a second French study [28] stated that these cases did not have KD but another disease that shared similar features, and they could be differentiated by a more severe presentation than KD, older age of onset and cardiac complications such as myocarditis. In the Spanish study, more than half of the pandemic group had positive SARS-Cov-2 exposure (PCR or serology) and 88.5% of the positive exposure cases fulfilled the diagnostic criteria for MIS-C. Hence, it can be inferred that the reported increase of KD cases in these countries were most likely due to KD-like MIS-C cases.

In this study, 30.8% of patients had a positive SARS-CoV-2 exposure. However, positive exposure does not signify a MIS-C diagnosis since, similarly to KD, it requires precise criteria to be fulfilled [29, 30]. We have considered the first three months of the pandemic, February to April 2020, as a period in which MIS-C had not yet been described or did not have precise diagnostic criteria and in which a MIS-C case may have been misdiagnosed as KD. Only 7 cases in the pandemic group were diagnosed during that period and only 2 of them had a positive exposure to SARS-CoV-2.

Even though there is some phenotypic overlap with MIS-C and KD, key features that distinguish the two diseases include: older age, higher incidence of gastrointestinal symptoms, myocardial dysfunction and higher levels of inflammatory markers in MIS-C [31]. In this study, children were younger than the average age expected for MIS-C, gastrointestinal symptoms were only present in about 30% of patients and its incidence did not increase in the pandemic group. There were no cases of myocardial dysfunction and inflammatory markers were not higher in the pandemic group.

Another hypothesis explaining such an increase in incidence is brought forth by J.A. Burney et al., who suggested that social behavior is associated with a KD trigger [13]. Through their study of the spaciotemporal distribution of KD during the pandemic, they discovered a shift of KD incidence proportionally increasing in neighborhoods with higher socioeconomic status, which in turn was associated with reduced time spent away from home during the shelter-in-place period. This led them to suggest that KD triggers were more likely found inside the home, which was a hypothesis that was also suggested by Ae et al. [30].

Our study and two other studies [15, 17] noted less inter-seasonal variation in incidence during the COVID-19 period. This observation further supports the hypothesis that studies that have taken into account data from pandemic periods of less than a year may have less accurate calculated incidences.

Regarding the clinical characteristics of the disease, we have found no change in distribution of sex, which has also been found in other studies [14, 17]. Although we have found no significant decrease in the age of onset, as did one of the Japanese studies [17], and we have found that the proportion of children under the age of 1 year did not vary, multiple other studies did not replicate our findings. Instead, they showed a greater proportion of KD incidence in children under 1 year of age and, inversely, a smaller proportion of children between 1 and 5 years of age [14, 16], thus finding a decrease in their median age [18, 31]. This phenomenon has been explained by older children changing lifestyles during the pandemic, such as lessened time outdoors and mask wearing, whereas younger children did not undergo as drastic lifestyle changes in comparison to before the pandemic [31]. Alternatively, Esmaeilzadeh H. et al. suggested that SARS-CoV-2 infection acts as a KD trigger more strongly in younger children than in those over 1 year old [18].

Globally, as our study has also found, no significant change in laboratory values were found between the two periods [13, 14, 18].

Coronary artery outcomes remained unchanged, as has been found in the other studies [13, 31].

As in our study, there have been no reported changes in the administered treatments during the two periods [14].

This study presents the usual limitations of a retrospective and single center study, such as limited generalizability in regard to other regions. Furthermore, the incidence of KD was calculated using estimates of which part of the Swiss population would refer to the University Hospital of Lausanne instead of another tertiary center. The cantonal registries on which we based our calculations provided populations aged 0 to 19 years. Therefore, to estimate the population aged 0 to 17 years, the calculations were based on the assumption that every age group were the same size and the total was adjusted accordingly.

This study also has multiple strengths. Firstly, to the best of our knowledge, it is the first prolonged European study to compare KD parameters before and during the COVID-19 pandemic, which involves epidemiology, clinical presentation, coronary outcomes and treatment. Secondly, the data collected covered 6 years, almost 3 of which during the COVID-19 pandemic. Finally, this study highlights the significance of genetic and ethnic susceptibility, as well as environmental and immunological factors in the pathogenesis of Kawasaki disease.

## Conclusions

There has been a significant increase in the incidence of Kawasaki disease in Switzerland during the COVID-19 pandemic, whereas studies done in Japan, South Korea and the United States have found a decrease in incidence. Differences in study methodologies, genetics, ethnicities, environments, microbiome-altering behaviors, sanitary measures and SARS-CoV-2 virus spread are factors that should be considered.

The clinical presentation, laboratory values and coronary outcomes have not significantly changed during the COVID-19 pandemic.

Further studies analyzing the differences between countries with increased incidence of KD could help better understand the relevance and roles of such factors and provide more insight into the etiologies of this particular disease.

## Data Availability

The datasets used and/or analyzed during the current study are available from the corresponding author on reasonable request.

## Abbreviations

KD: Kawasaki disease
IVIG: intravenous immunoglobulins
ASA: acetylsalicylic acid
COVID-19: coronavirus disease 2019
MIS-C: multisystem inflammatory syndrome in children
AHA: American Heart Association
CAI: coronary artery involvement
RCA: right coronary artery
LMCA: left main coronary artery
LAD: left anterior descending artery
Cx: left circumflex artery
CS: corticosteroids
